# Delayed egg-laying in Red-backed Shrike *Lanius collurio* in relation to increased rainfall in east-central Poland

**DOI:** 10.1007/s00484-023-02450-2

**Published:** 2023-03-07

**Authors:** Artur Golawski, Sylwia Golawska

**Affiliations:** Faculty of Sciences, Siedlce University of Natural Sciences and Humanities, Prusa 14, 08-110 Siedlce, Poland

**Keywords:** Clutch phenology, Clutch size, Egg size, Long-term study, Weather factors

## Abstract

Climate change has affected the breeding parameters of many animal species. In birds, most studies have focused on the effects of temperature on clutch phenology and clutch size. The long-term influence of other weather factors, including rainfall, on breeding parameters have been analysed much less often. Based on a 23-year dataset and 308 broods, we documented shifts in the timing of breeding, clutch size and mean egg volume in a long-distance migrant, the Red-backed Shrike *Lanius collurio*, from a central European population. We found a 5-day shift towards delayed breeding, but no differences in brood size or egg volume during those 23 years. The GLM analysis showed that the mean May temperature had a positive influence on the clutch initiation date, whereas the number of days with rain delayed laying. During the period 1999–2021, there was no change in the mean May temperature, but total precipitation and the number of days with rain in May increased. Thus, delayed nesting in this population was probably due to the increase in rainfall during this period. Our results provide a rare example of delayed nesting in birds in recent years. Predicted changes in the climate make it difficult to assess the long-term impact of global warming on the viability of Red-backed Shrike populations in east-central Poland.

**Supplementary Information:**

The online version contains supplementary material available at 10.1007/s00484-023-02450-2.

## Introduction

In recent decades, the Earth's climate has undergone changes, manifested mainly by increases in ambient temperatures, changes in rainfall patterns and the occurrence of extreme weather events (Houghton 2015). Such climate changes are having serious consequences for various species of plants and animals (Bellard et al. 2012; Gray and Brady 2016; Radchuk et al. 2019). The earlier arrivals of many migratory birds in their breeding areas and earlier laying dates during the last 20-30 years have been described over vast geographical areas (Tryjanowski et al. 2002; Both et al. 2004; Shipley et al. 2020). Changes in the breeding phenology in birds and the subsequent effects on their broods, especially among insectivorous species, are the consequence of altered access to food resulting from climate changes (Both et al. 2004; Charmantier et al. 2008). Most analyses have implicitly or explicitly assumed that temperature is the major driver of changes in breeding phenology and brood parameters in birds (Charmantier et al. 2008; Møller et al. 2010). Other studies have shown that higher temperatures favour earlier nesting, with the shift to earlier first-egg laying dates in some cases being more than 5 days in 10 years (Dunn and Winkler 1999; Halupka et al. 2020). Generally, early breeders tend to have larger clutches (Lack 1968; Dunn and Møller 2014) and are more likely to double brood (Bulluck et al. 2013; Townsend et al. 2013), but this is not always the case, probably because of the mismatch with the period of food abundance (Laaksonen et al. 2006; McDermott and Degroote 2016; Halupka et al. 2020). Some published data also show that the eggs of the species analysed became progressively smaller as a result of the mismatch between the dates food abundance and optimal egg formation by the female (Tryjanowski et al. 2004; Potti 2008). Because egg size affects juvenile survival, it is a very important breeding factor (Krist 2011).

The long-term effects of other weather factors on breeding parameters have been analysed far less often, one of these being rainfall (e.g. Laaksonen et al. 2006; Drake and Martin 2020). Climate change is manifested, for instance, by shifts in the intensity and duration of rainfall (Dore 2005). This appears to be of considerable significance for species inhabiting arid regions (Schneider and Griesser 2009; Cavalcanti et al. 2016) and tropical regions with a dry season (Brawn et al. 2017; Shaw 2017), as it stimulates plant growth and enhances food availability for birds at this time, thus improving female condition prior to egg laying (Oppel et al. 2014). On the other hand, the few published long-term data on breeding parameters in conjunction with rainfall from the temperate zone are not as convincing as in the case of air temperature. One such example demonstrated that local rainfall was an important negative driver of breeding phenology in Tree Swallows *Tachycineta bicolor*, although the productivity cost was minimal (Drake and Martin 2020). But in several other North American species, rainfall had no influence whatsoever on either clutch initiation dates or clutch sizes (Bowers et al. 2016; Drake and Martin 2018). Despite positive suggestions regarding the dominant part played by temperature in advancing nesting (Crick and Sparks 1999), some authors, e.g. Irons et al. (2017), state that it is actually rainfall that exerts a stronger influence on breeding phenology than temperature.

One of the most powerful approaches to inferring ecological effects of climate and climate change is to track phenological changes of local populations (Irons et al. 2017), and given the high annual variability of ecological studies, long-term research is key to discerning patterns and responses to global changes (Lindenmayer et al. 2012). The present study examines the breeding phenology and certain breeding parameters in Red-backed Shrikes *Lanius collurio* nesting in east-central Poland in the context of climate factors. This species was also the subject of similar studies involving temperature and rainfall in both the Czech Republic (Hušek and Adamík 2008) and western Poland (Tryjanowski 2002; Tryjanowski et al. 2004), but their results were inconclusive. The aim of the present study was to demonstrate the phenological trends relating to nesting, clutch size and mean egg volume in Red-backed Shrike broods during the last 23 years as affected by two basic climatic factors, i.e. air temperature and rainfall.

## Material and methods

### Study species

The Red-backed Shrike is a small passerine bird species widely distributed in Europe and western Asia, with population estimates ranging from 24 to 48 million breeding pairs, and because this species has a large population, it has been evaluated as being of Least Concern, although its numbers do appear to be decreasing (BirdLife International 2021). It is a long-distance migrant, which arrives at its breeding grounds from Africa in April/May (Harris and Franklin 2000). In eastern Poland, the majority of the population inhabits agricultural landscapes, breeding on the edge of woods, in clumps of trees, in orchards and near villages (Golawski and Meissner 2008). The breeding season usually starts in mid-May and extends into August. Three to seven eggs are laid and incubated for 14 days. The nestlings remain in the nest for the next 14 days, and fledglings stay around the nest for another two weeks or so (Harris and Franklin 2000). This species is normally single-brooded, but in case of first-brood failure, replacement clutches are laid regularly (Antczak et al. 2009). The Red-backed Shrike is a mainly insectivorous species (Tryjanowski et al. 2003; Golawski 2006).

### Study site and data collection

**Fig. 1:**
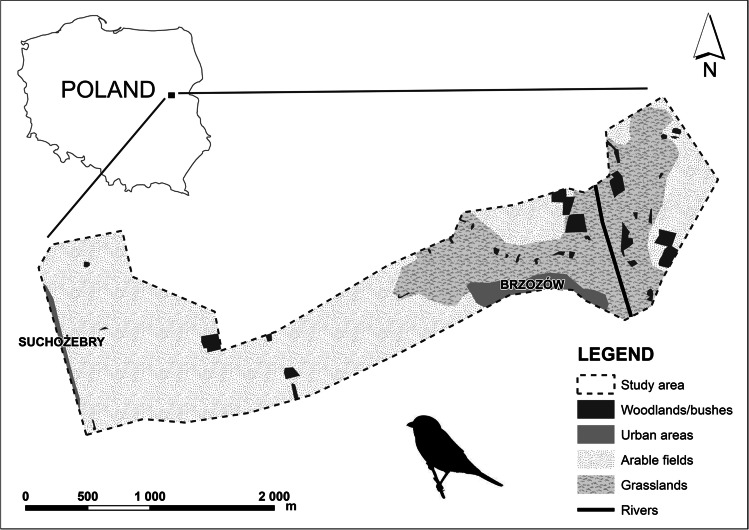
Map of the study area

The survey was carried out on ca 500 ha of the agricultural landscape around the town of Siedlce in eastern Poland (52.14° N, 21.93°E, Fig. 1), which has a temperate transitional climate (Degirmendzic et al. 2004). The study area abounded in meadows and pastures, subdivided by barbed wire fences, and there were scattered bushes and trees. Part of the research area was situated along a railway line with plenty of shrubs suitable as shrike nesting sites.

The study was carried out in the 1999-2003 and 2012–2021 breeding seasons, a period spanning 23 years. In each season, observations started in early May, when the first shrikes arrived, and ended in July/August, when the last pairs departed with their young. We searched for Red-backed Shrike nests, checking possible locations favourable for nesting and observing the birds’ behaviour, e.g. a male feeding the incubating female. We found between 10 and 59 (mean ± SD = 29.5 ± 15.3) nests each year. As most nests were found during egg laying or the early phase of incubation, calculating the clutch initiation date was straightforward; incubation advancement was based on the water test of the eggs (Wesołowski 1986). About 30% nests were found with nestlings. In these cases, the nestlings were weighed and their age estimated according to the age-body mass relationship, described for this species on the basis of data gathered in natural conditions (Diehl 1971). We assumed that Red-backed Shrikes lay one egg per day, begin incubating from the day when the penultimate egg is laid and continue to do so for 14 days (Harris and Franklin 2000). The detected nests were checked every 4–5 days to minimize the impact of the observer’s intrusion on nest survivorship (Tryjanowski and Kuzniak 1999; Golawski and Zduniak 2022). We measured eggs only in complete clutches (N = 142). The maximum length and breadth were measured with sliding callipers to the nearest 0.1 mm (all eggs measured by AG). We calculated the egg volume index (V) from the length (L) and breadth (B) using the formula re-scaled for the Red-backed Shrike: V = 0.5322 LB^2^ (Surmacki et al. 2006). The analysis does not include clutches in which brood advancement could not be accurately calculated.

Our records contain data relating to both first and replacement broods. As the inclusion of replacement broods is likely to influence the phenological statistics (Antczak et al. 2009), we defined first broods as those initiated before 10 June (for an identical approach, see Tryjanowski 2002). Our dataset thus consists of 308 broods out of the total of 442 recorded. On average, 40 pairs of shrikes nested in the study area each year (SD = 2.5, Range 36-44 pairs, n = 15 years), and the population showed no significant changes in numbers (Spearman rank correlation, R = -0.23, P = 0.418, n = 15).

### Meteorological data

We used local weather data (Electronic supplementary material) to investigate the effect of climatic factors on the timing of breeding, clutch size and egg volume. Like Hušek and Adamík (2008) and Hušek et al. (2009), we used the weather data for the month of May, because it is during that month that Red-backed Shrikes arrive in east-central Poland and when most birds make their first breeding attempts (Antczak et al. 2009). The weather in May should thus affect laying dates and breeding parameters. We obtained the local weather data of interest to us, i.e. mean diurnal temperatures (in °C) in May, total precipitation (monthly sums in mm) and the number of days with rain in May, from http://www.tutiempo.net for the nearest meteorological station in Siedlce (a town 10 km south of the study area).

### Statistical analysis

We used General Linear Models (GLM) with normal distribution and identity link functions to identify the factors affecting the Red-backed Shrike’s breeding parameters. We performed 3 analyses, where the dependent variables in the models were: 1) date of clutch initiation (when the first egg was laid), 2) clutch size, 3) mean volume of the eggs in a clutch. Mean values of all egg characteristics in the clutch were used as unit observations to avoid pseudoreplication (Lessells and Boag 1987). The explanatory variables used in all the models were weather factors in May: mean temperature, total precipitation and number of days with rain. Because population size could impact on nest detection – there is a higher probability of observing earlier clutch initiation when the population is larger (Tryjanowski and Sparks 2001; Hušek et al. 2009) – we used the number of nests in a year as a covariate. Another aspect to be taken into account is that the level of philopatry in the population studied is very low (Tryjanowski et al. 2007), so the probability that the same birds were included several times is rather low. The last explanatory variable used was the year of the study (1999-2002, 2012-2021). In all the GLMs, the interaction between temperature and the number of days with rain was introduced in the initial parameterization, but subsequently removed from the models because it was not significant in any of the analyses.

The explanatory variables were tested for multicollinearity by examining the Variance Inflation Factor (VIF) (Quinn and Keough 2002). When VIF > 5, we discarded the variable from the analysis; one variable – total precipitation – was above the critical value, so we excluded it from the analysis. As not all breeding metrics were available for all broods, the sample sizes varied. Only those results with a probability of α ≤ 0.05 were assumed to be statistically significant. The analysis was performed in Statistica 12.0 (Statsoft 2014).

## Results

### Long-term changes in meteorological data

In east-central Poland during the study period (1999–2021), the May temperature showed no changes (linear regressions: F_1,21_ = 0.36, R^2^ = 0.02, P = 0.556; slope ± SE = -0.129 ± 0.216). However, total precipitation in May exhibited a positive trend (F_1,21_ = 4.35, R^2^ = 0.17, P = 0.049; slope ± SE = 0.414 ± 0.199) over the study period. Similarly, in the same period, the number of days with precipitation in May increased (F_1,21_ = 4.89, R^2^ = 0.19, P = 0.038; slope ± SE = 0.434 ± 0.199) (Electronic supplementary material).

### Effects of weather on breeding parameters

**Fig. 2:**
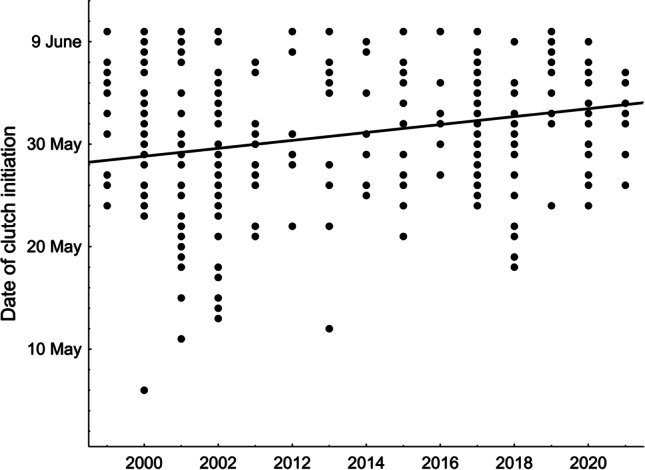
Temporal trends (1999–2021) in the clutch initiation date in the Red-backed Shrike *Lanius collurio* in east-central Poland

**Fig. 3:**
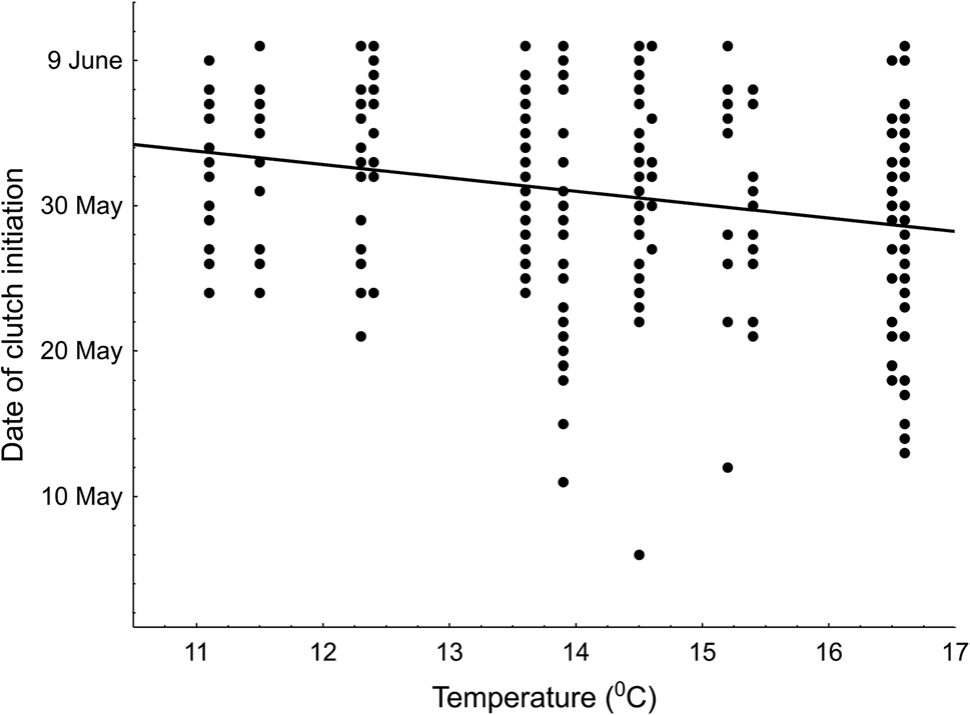
Relationships between the clutch initiation date in the Red-backed Shrike *Lanius collurio* and mean May temperature in east-central Poland

**Fig. 4:**
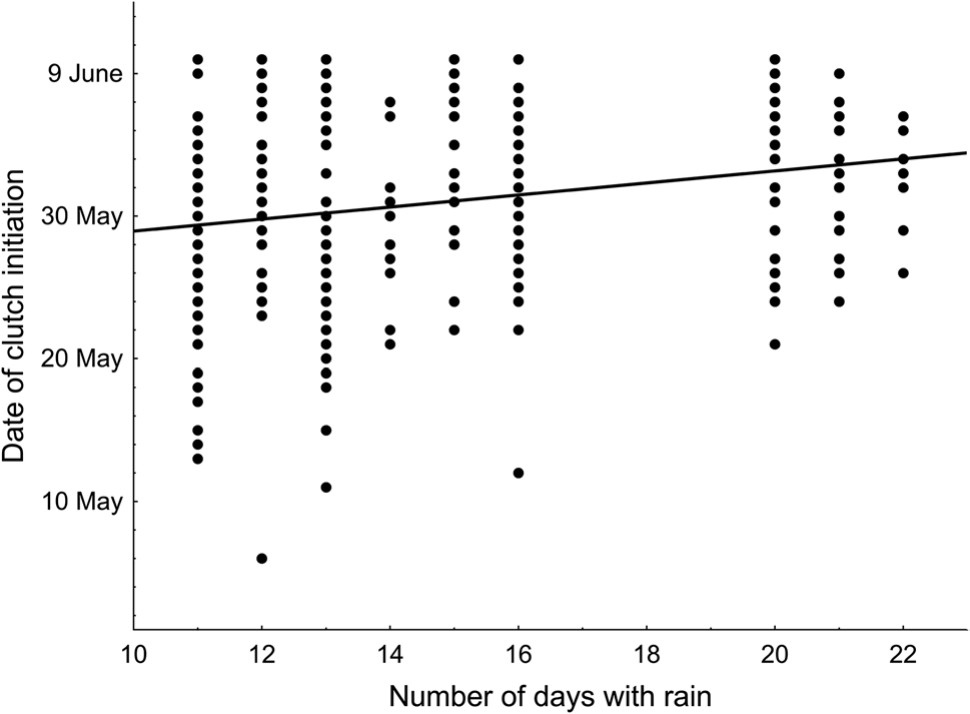
Relationships between the clutch initiation date in the Red-backed Shrike *Lanius collurio* and the number of days with rain in May in east-central Poland

The mean clutch initiation date in our Red-backed Shrikes was 31 May (SD = 6.6 days, range 6 May-10 June, n = 308 broods) and the date differed with respect to the factors analysed (GLM, F_4,303_ = 10.33, p < 0.001, R^2^ = 0.12, Table 1). In subsequent years, the shrikes initiated their clutches later and later (Fig. 2). Analysis of the trend showed that between 1999 and 2021 the shrikes nested 5.03 days later (2.2 days/10 years). The mean May temperature had a significant influence on the clutch initiation date; when temperatures were higher, the shrikes nested earlier (Fig. 3). In contrast, clutch initiation was delayed if there was a large number of days with rain in May (Fig. 4). The number of clutches in a given year did not affect the clutch initiation date (Table 1).

**Table 1:** GLM of the predictors (mean air temperature in May, number of days with rain in May, number of clutches in a given year, year of the study) affecting the clutch initiation date, clutch size and mean egg volume in a clutch in the Red-backed Shrike *Lanius collurio*, 1999-2021

Parameter	Estimate	SE	*F*	*P*
Date of clutch initiation (n = 308 clutches)				
Intercept			52.24	<0.001
**Temperature**	**-0.265**	**0.083**	**10.27**	**0.001**
**Days with rain**	**-0.237**	**0.109**	**4.78**	**0.030**
Number of clutches	-0.109	0.077	2.01	0.157
**Year**	**0.282**	**0.080**	**12.47**	**<0.001**
Clutch size (n = 235 clutches)				
Intercept			20.94	<0.001
Temperature	0.150	0.100	2.23	0.137
Days with rain	0.091	0.131	0.49	0.483
Number of clutches	0.016	0.130	0.03	0.864
Year	-0.085	0.096	0.78	0.377
Mean egg volume (n = 142 clutches)				
Intercept			81.49	<0.001
Temperature	-0.205	0.126	2.66	0.105
Days with rain	0.021	0.167	0.02	0.898
Number of clutches	0.075	0.130	0.33	0.564
Year	-0.074	0.131	0.32	0.571

The mean clutch size was 5.5 eggs (SD = 0.7, Range 3-7, n = 235 clutches) and did not differ with respect to the factors analysed (GLM, F_4,230_ = 1.08, p = 0.369, R^2^ = 0.02, Table 1).

The mean egg volume in the clutches was 3.19 cm^3^ (SD = 0.25, Range 2.62-3.80, n = 142 clutches), and this, too, did not differ with respect to the factors analysed (GLM, F_4,137_ = 1.27, p = 0.285, R^2^ = 0.04, Table 1).

## Discussion

We found that the total rainfall and the number of days with rain in May in east-central Poland increased significantly between 1999 and 2021, whereas no such trend as regards air temperature was discernible. Testing the influence of these two climatic variables revealed that the number of days with rain in May significantly delayed clutch initiation in Red-backed Shrikes. Over the 23 years of the study, this delay in nesting became as long as 5 days, the most probable cause of this in our opinion being the increase in rainfall (the greater number of days with rain in May) during that period. In contrast, higher May temperatures favoured the earlier nesting of the birds. The climatic variables under scrutiny here did not affect either clutch size or egg volume.

Although many bird species have advanced their start of egg laying and changed their breeding parameters in recent years (Costantini et al. 2010; Goodenough et al. 2010), some data indicate delayed nesting (e.g. Bates et al. 2022). In particular, changing brood parameters are not so clear-cut in long-distance migrants than in short-distance migrants, because they may be constrained by factors acting during the winter and migration periods (Tryjanowski et al. 2002; Laaksonen et al. 2006; Kallander et al. 2017). This probably applies to the Red-backed Shrike as well, because the influence of climatic factors on laying dates and reproductive parameters is highly ambiguous. In the Czech Republic, Hušek and Adamík (2008) found a 3- to 4-day shift towards earlier breeding and an increase in brood size by approximately 0.3 nestlings in the period 1964–2004. In western Poland in 1971–2002, Red-backed-Shrikes arrived at their breeding grounds significantly earlier, and the arrival date was correlated with the earliest first-egg date (Tryjanowski et al. 2004). On the other hand, the nesting dates for two periods (1905-1935 vs. 1985–1999) did not show any difference in the time of laying or clutch size in Poland (Tryjanowski 2002). Also, no significant correlation between the arrival date and the research period were recorded in north-western Croatia between 1991 and 2016 (Dolenec 2018). In contrast, the data we give in this paper point to quite a distinct delay in the start of nesting by Red-backed Shrikes in east-central Poland in the last 23 years, albeit without any trends whatsoever in clutch size or the mean egg volume in a clutch.

The effect of climate factors is fairly clearly reflected by changes in the clutch initiation date and breeding parameters. Our study has shown that higher May temperatures induce earlier nesting in Red-backed Shrikes, and identical relationships have also been found elsewhere (Matyjasiak 1995; Hušek and Adamík 2008; Hušek et al. 2009). The second dependence found in east-central Poland was the rainfall-induced delay in the onset of breeding, which also concurs with earlier studies (Hušek and Adamík 2008; Metzmacher and Van Nieuwenhuise 2012). Red-backed Shrikes appear to be particularly sensitive to rainfall (Tryjanowski et al. 2003). In east-central Poland, egg volume repeatability was related to the total rainfall immediately before egg laying (Golawski and Mitrus 2018) and breeding success was significantly dependent on the number of days with rain (Golawski and Golawska 2019). The partial loss of nestlings was also related to the total rainfall (Golawski 2006).

The energy status of laying females may deteriorate during inclement weather with low temperatures and rainfall because of the higher energetic cost of thermoregulation (Stevenson and Bryant 2000), and/or the reduced availability of food, especially for insectivorous species, resulting from delays in insect development and also their activity (Vicens and Bosch 2000; Arbeiter et al. 2016). A reduced food supply for laying females affects their condition, which in turn may delay nesting and result in poorer breeding parameters (Tryjanowski et al. 2004). The Red-backed Shrike is a mainly insectivorous species (Tryjanowski et al. 2003; Golawski 2006), so limited access to food can significantly affect the birds’ condition. Rainfall could therefore have driven the delay in clutch initiation in east-central Poland. Moreover, birds adjust their breeding season to the greatest availability of food when they are feeding their young (Both et al. 2006). A cooler and rainier spring means that insects develop later, especially orthopterans, which are a very important component of the shrikes’ diet in this region (Morelli et al. 2016; Golawski and Kondera 2023), so this may be a reason for the delay in clutch initiation. Delayed nesting can have serious consequences for birds, because females laying later in a given breeding season produce smaller clutches (Lack 1968); there will also be fewer re-nesting opportunities (Halupka et al. 2008). Red-backed Shrikes can re-nest if the first brood is lost, but this depends on the period of the breeding season when that loss occurred (Antczak et al. 2009), so later nesting can make a big difference to them.

Rainfall can also affect gonad development in birds. This depends on the light intensity (Dawson 2015), which during rainfall (when the sky is overcast) is less than on sunny days (Mumby et al. 2001; La and Park 2016) and the gonads then develop more slowly. In consequence, the birds' breeding season is delayed. However, we were unable to evaluate this dependence as we had no light intensity data for the study area to hand.

On the other hand, the number of eggs and their volume was independent of weather factors. Male Red-backed Shrikes feed their females before egg laying begins and set up larders where they store prey items, later to be consumed by the females (Yosef and Pinshow 2005). Such behaviour by the male to some extent compensates for the limited amount of food available to the female during poor weather, thereby enabling her to remain in relatively good condition, as demonstrated earlier in this region (Golawski et al. 2020). Moreover, the study area included habitats with extensive agriculture which are abundant in the shrikes’ potential prey (Golawski and Golawska 2008).

## Conclusion

Our research shows that delayed clutch initiation by Red-backed Shrikes in the last 23 years has probably been a consequence of the increasingly frequent rainfall in this period. At the same time, air temperature did not display a clear trend. However, we emphasize that that our data are correlative: this does not mean that rainfall patterns are the underlying cause of changes in clutch metrics, merely that they are associated with each other. This is a good example of the fact that not only temperature but also rainfall can change the time of clutch initiation. Hence, predicting the long-term effects of global warming on the viability of east-central Polish populations of the Red-backed Shrike is difficult.

## Supplementary information

Fig. S1 Relationship between weather factors and year: a) mean May air temperature, b) total precipitation, c) number of days with rain (data for meteorological station in Siedlce, east-central Poland) (DOCX 261 kb)

## Data Availability

The datasets generated and/or analysed during the current study are available from the corresponding author on reasonable request
