# Supplementary material for: Delayed egg-laying in Red-backed Shrike Lanius collurio in relation to increased rainfall in east-central Poland
Source: Int J Biometeorol. 2023 Mar 7;67(4):717–24. doi: 10.1007/s00484-023-02450-2 (PMC10070303; doi:10.1007/s00484-023-02450-2)

Delayed egg-laying in Red-backed Shrike *Lanius collurio* in relation to increased rainfall in east-central Poland

International Journal of Biometeorology

Artur Golawski^*^, Sylwia Golawska

Siedlce University of Natural Sciences and Humanities, Faculty of Sciences, Prusa 14, 08-110 Siedlce, Poland

*Corresponding author: [artur.golawski@uph.edu.pl](mailto:artur.golawski@uph.edu.pl)

**Fig. S1** Relationship between weather factors and year: a) mean May air temperature, b) total precipitation, c) number of days with rain (data for meteorological station in Siedlce, east-central Poland)


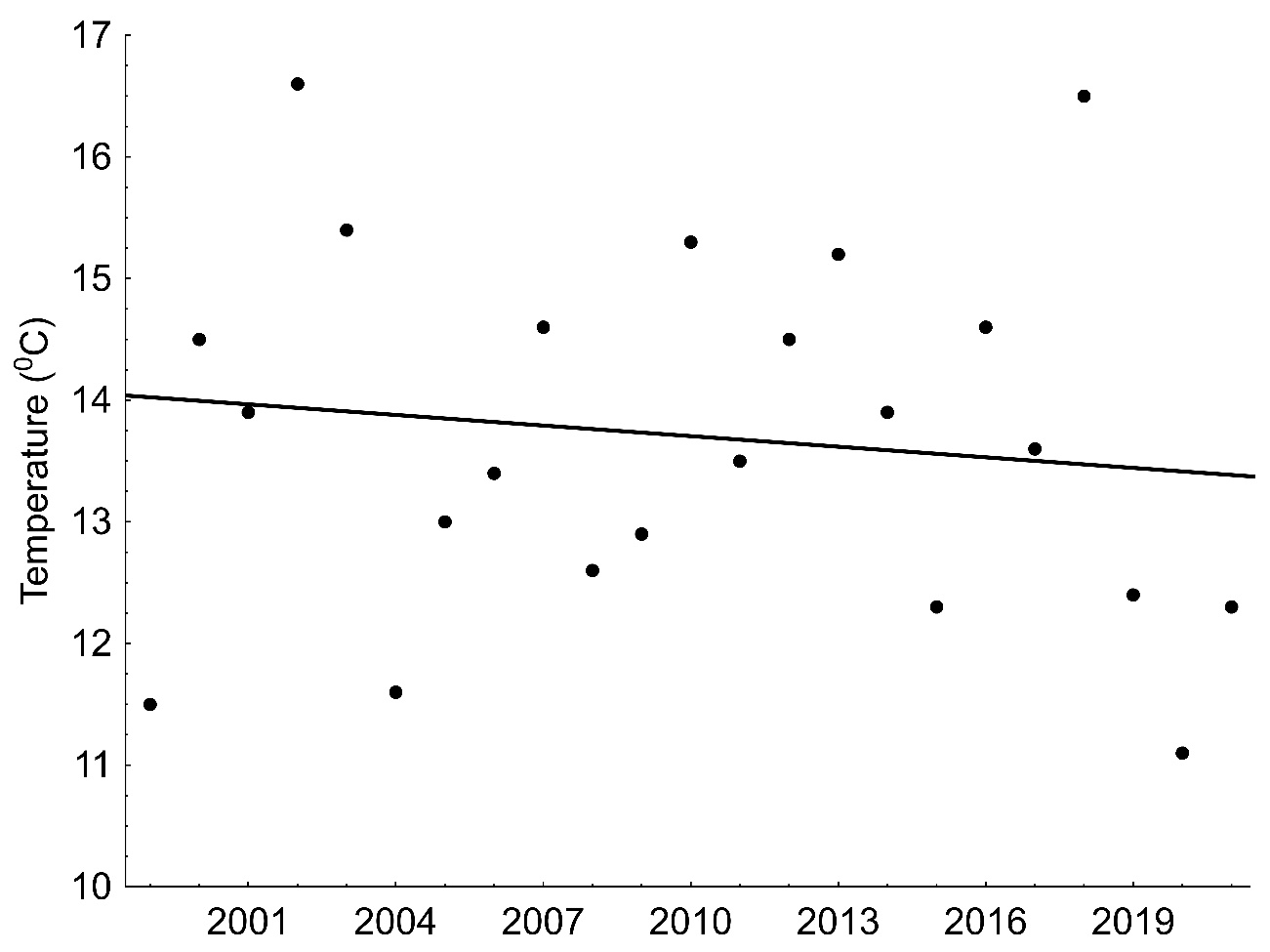


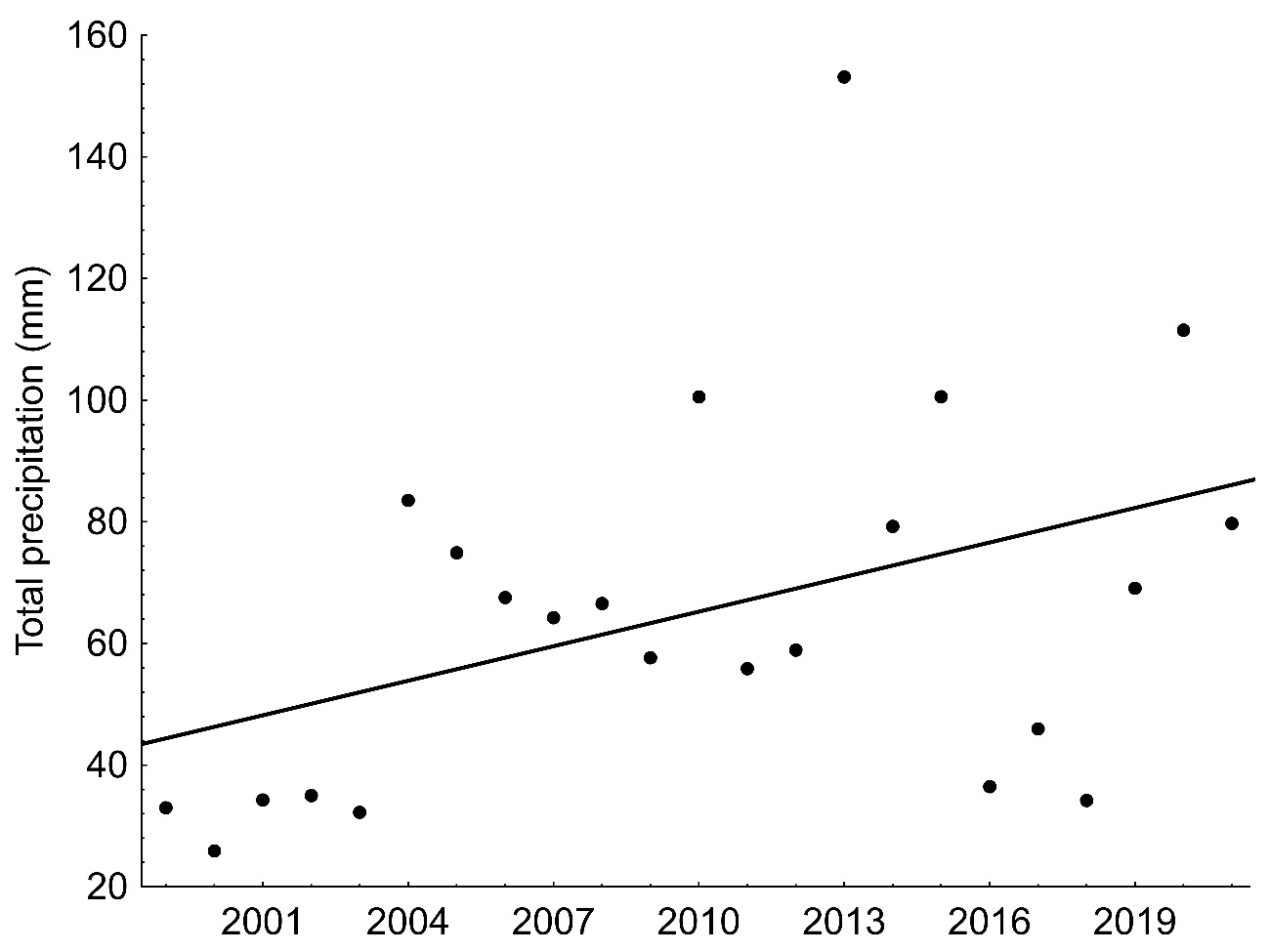


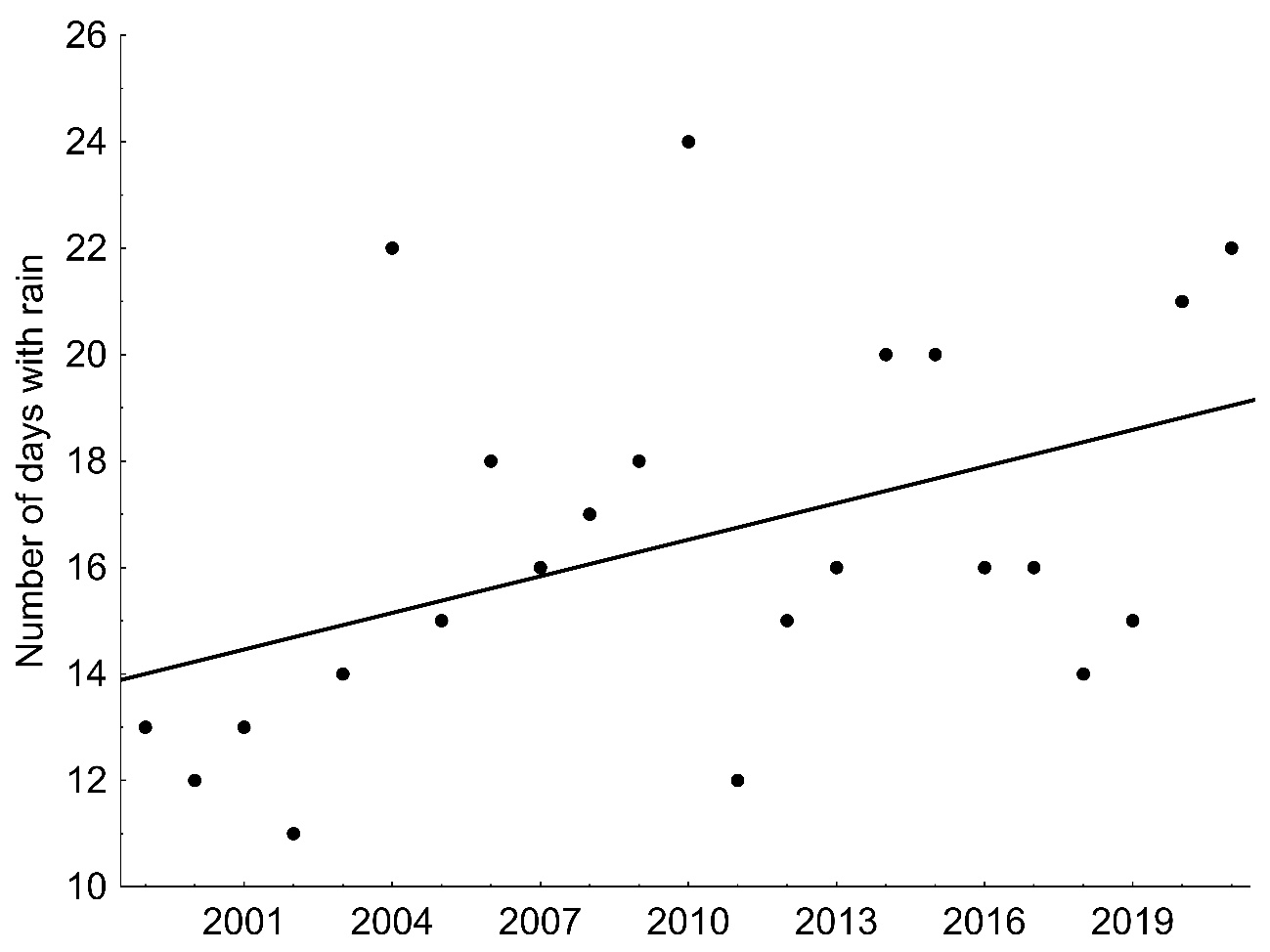

Supplement: Supplementary file 1 — Relationship between weather factors and year: a) mean May air temperature, b) total precipitation, c) number of days with rain (data for meteorological station in Siedlce, east-central Poland) (DOCX 261 kb) [file 484_2023_2450_MOESM1_ESM.docx]
